# Effectiveness of school vision screening by teachers – a systematic review and meta-analysis

**DOI:** 10.1186/s12889-026-27333-0

**Published:** 2026-04-20

**Authors:** Ujwala Umesh, Krithica Srinivasan, Sharath S Sherigar, Vignesh Prabhu

**Affiliations:** Department of Optometry, Manipal College of Health Professions, Manipal Academy of Higher Education, Manipal, India

**Keywords:** Vision screening, School teacher, Eye care professionals, Sensitivity, Specificity

## Abstract

**Background:**

Visual impairment among school children is a prevalent concern that can affect their academic performance, intellectual, and interpersonal growth. Due to a lack of eye care professionals, school teachers were commonly involved in vision screening programs, and their effectiveness has been reported by a few studies with some methodological variations. Therefore, this systematic review aims to assess the effectiveness of teacher-led school vision screening programs in comparison with vision screening done by eyecare professionals.

**Methods:**

The Preferred Reporting Items for Systematic Reviews and Meta-analyses (PRISMA) standards were followed for conducting the review. MEDLINE, Scopus, and Web of Science were the databases used to search for original research articles using the keywords “vision screening,” “school children,” “teacher,” or related keywords. All quantitative studies were considered in this systematic review, while research articles involving a screening population greater than 18 years old were excluded. The meta-analysis was conducted utilizing the random effects model in the MetaDTA program.

**Results:**

Based on the eligibility criteria, nine original research studies were taken into consideration for this systematic review. The summary receiver operating characteristics (SROC) curve analysis was performed. Two studies were excluded from the reanalysis due to poor sensitivity values and wider confidence intervals, indicating greater variability in sensitivity estimation. Pooled sensitivity and specificity of teacher conducted vision screening from seven studies were estimated to be 0.70 (95% CI [0.57, 0.80]) and 0.96 (95% CI [0.91, 0.98]), respectively.

**Conclusion:**

The results of this systematic review indicate that teacher conducted vision screening provides satisfactory results in comparison with vision screening done by eye care professionals. Therefore, school teachers can be a potential workforce for early identification of visual impairment and contribute to lowering the disease burden.

**Supplementary Information:**

The online version contains supplementary material available at 10.1186/s12889-026-27333-0.

## Introduction

 Globally, refractive error is the most common cause of visual impairment (VI), with a prevalence of 77.20%, and is a primary cause of preventable blindness [1, 2]. The World Health Organization (WHO) launched VISION 2020, an initiative to end preventable blindness globally due to the enormous burden of VI, its causes, and its visual consequences [3, 4]. According to the WHO’s estimation at the beginning of the VISION 2020 program, approximately 19 million children had visual impairment, of which 1.4 million children had irreversible blindness, and it is predicted that 50% of all cases of childhood blindness could be averted [2, 5].

Uncorrected refractive error (URE) lowers an individual’s quality of life and may also affect their ability to learn and pursue specific professional options [6]. School based vision screening is a widely implemented method for early identification of visual impairment, as it allows screening of large numbers of children. Globally, the average number of Ophthalmologists per million population ranges from 3.7 to 76.2 in lower and higher income countries, respectively [7]. Additionally, on average there is one optometrist for every 23,200 people worldwide [8]. To overcome this shortage of eye care professionals, school teachers can be involved in eye screening programs.

In school eye screening (SES) programs, teachers were the primary examiner to screen the vision in children and help in the early detection of potentially blinding disorders [9]. The teacher’s experience in interacting with children and the initial availability of teachers in schools are the primary factors that led to the inclusion of teachers in children’s vision screening [10]. The benefit of engaging teachers in school vision screening programs can be more effective in encouraging children and parents to visit an optometrist, and they will be more helpful in encouraging children to use glasses regularly [11]. The SES program approach has been successfully used in the school setting and has developed over time to screen for refractive error and other ocular abnormalities [9]. Numerous studies have evaluated the effectiveness and efficiency of SES programs. The SES programs facilitate the screening of large populations in school settings, thereby reducing the workload for eye care professionals [12]. Results of school vision screening studies have shown equivocal evidence regarding the effectiveness of such programs. Therefore, this systematic review and meta-analysis aim to assess the effectiveness of school vision screening conducted by teachers against vision screening conducted by eye care professionals.

## Methodology

This systematic review was conducted following the Preferred Reporting Items for Systematic Reviews and Meta-analyses (PRISMA) recommendations. The systematic review was registered in PROSPERO on March 8, 2023, with registration number CRD42023402916, before the review began [13].

### Review question

PICO (Problem, Intervention, Comparison, Outcome) was defined before the literature search.

The review question was “To estimate the effectiveness of school vision screening conducted by trained school teachers in comparison with vision screening conducted by eye care professionals.” The systematic review aimed to explore the sensitivity and specificity of vision screening conducted by teachers for school children compared to that of vision screening conducted by eye care professionals. The outcomes for vision screening were compared between vision screening conducted by school teachers and vision screening conducted by eye care professionals (reference standard). In this review, vision screening conducted by trained eye care professionals such as ophthalmologists, optometrists, vision technicians, and primary eye care workers was used as the reference standard for comparison with screenings conducted by teachers.

### Search strategy

The relevant literature was searched in MEDLINE, Scopus, and Web of Science databases for studies published through April 2023. The search terms included “vision screening”, “school”, “children”, “teachers”, “eye care professionals”, “sensitivity”, and “specificity”. When necessary, the terms were separated during the search using the Boolean operators “OR,” “AND,” and “NOT,” as well as the Medical Subject Headings (MeSH). Various search strategies were employed, and the number of results for each strategy is given in the Supplementary material. Mendeley software (version 1.19.5) was used to import and manage each reference.

### Inclusion and exclusion criteria

The studies were included if the published literature screened children by trained school teachers, the research articles comparing teachers vision screening accuracy values to those of vision screening accuracy values of eye care professionals in school-based vision screening, where the screened population is under the age of 18 years and were included irrespective of the study region. The study articles were excluded if they were qualitative research, published in a language other than English, involved comparisons between vision screenings conducted by teachers and comprehensive eye examinations, or presented outcomes based on follow-up vision screening assessments conducted by school teachers.

### Study selection

Using Mendeley software, duplicate articles were eliminated following the search, and the remaining papers were subsequently examined for eligibility. The systematic review panel consisted of four reviewers. Two reviewers separately evaluated the titles and abstracts of the identified articles. A third and fourth independent reviewers helped to clarify any disagreements between the two reviewers. Two reviewers assessed the eligibility of the full-text articles based on the inclusion and exclusion criteria. Articles fulfilling the eligibility criteria were reviewed, and data were extracted from the primary reviewer and secondary reviewer.

### Data extraction

The primary and secondary reviewers extracted the data, and disagreements were resolved with the help of the third and fourth reviewers. The study title, year of publication, study authors, sample size, participant age, number of true negatives, number of true positives, number of false negatives, number of false positives, sensitivity, and specificity were extracted from all included studies. All the extracted information was managed in Microsoft Excel (version 2302; build 16130.20332).

### Statistical analysis

The statistical analysis was performed utilizing the MetaDTA program (v 2.01) [14]. This web-based tool is used for meta-analyses of studies on diagnostic accuracy. To assess a test’s performance, this tool synthesizes sensitivity and specificity results from various studies. Since the sensitivity and specificity values from each study use different criteria, which may lead to heterogeneity in pooled estimates, MetaDTA based on a random effects model was used to plot the summary receiver operating characteristics (SROC) curve. The SROC is a statistical method that is used to evaluate and summarize the performance of a diagnostic test across multiple studies [15]. The findings are displayed as an SROC curve, which provides a pooled estimate of the sensitivity and specificity values. It can also be used to provide sensitivity analysis by repeatedly analysing the effects of removing studies from the analysis. The Review Manager program (RevMan version 5.4.1) was used to determine the true positive (TP), true negative (TN), false negative (FN), and false positive (FP) values for the studies where TP, TN, FN, and FP values were not provided.

### Quality assessment

The Agency for Healthcare Research and Quality (AHRQ) was used to assess the quality of the included cross-sectional studies (19). With the help of the AHRQ tool, which consists of an 11-item checklist, the quality of all included cross-sectional studies was evaluated. If an item was marked as “Unclear” or “No,” it received a score of “0,” and if it was responded “Yes,” it received a score of “1.” The article’s quality was rated as low if the score was between 0 and 3, moderate if the score was between 4 and 7, and high if the score was between 8 and 11, as per the criteria [16].

## Results

### Identification and selection of the articles for the systematic review

**Fig. 1:**
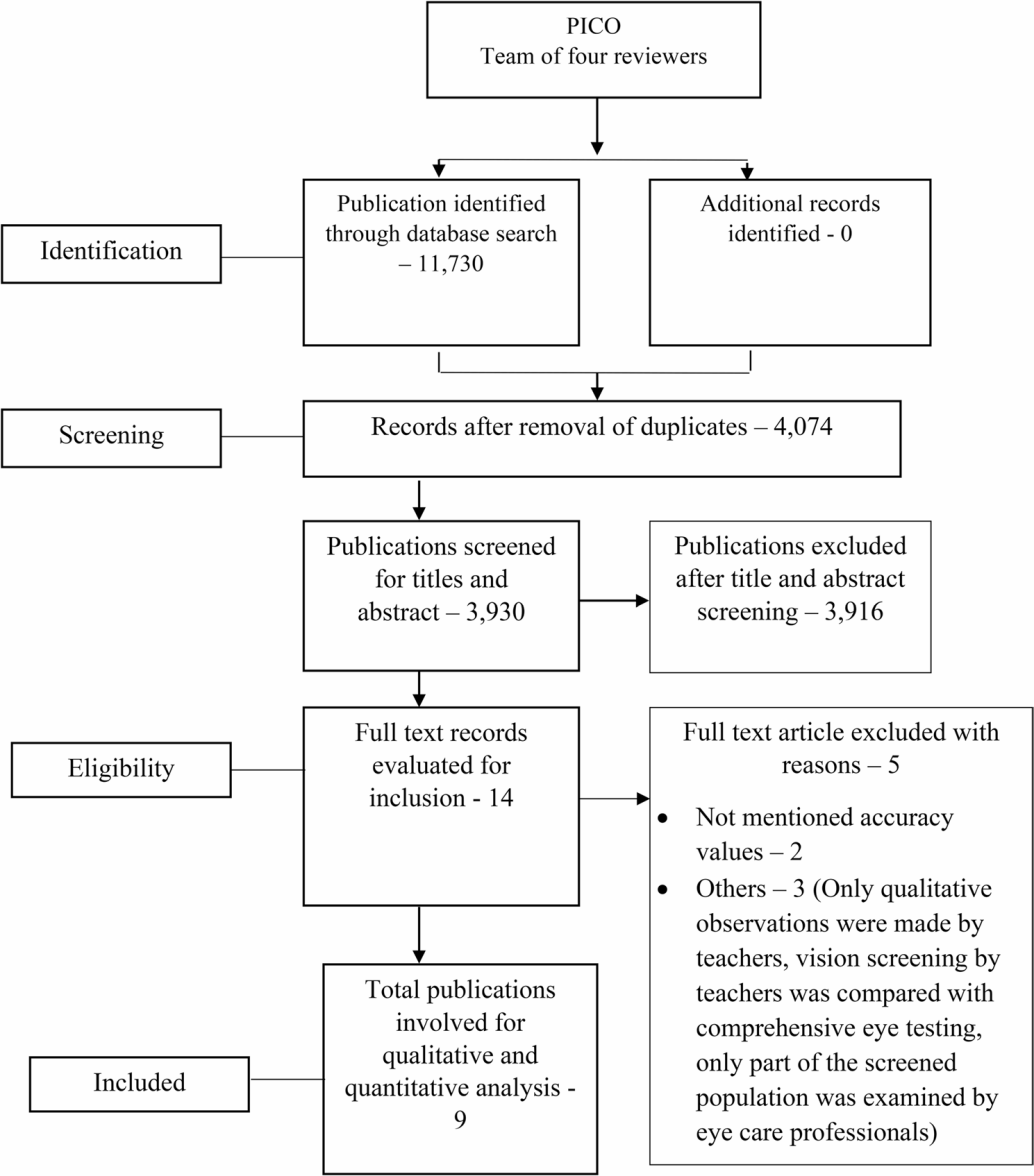
Flowchart of the systematic literature search process according to PRISMA. Flow chart describing the selection process and number of articles included and excluded in the stage of the systematic review process according to PRISMA guidelines

Overall, 11,730 documents were found in the search through MEDLINE, Scopus, and Web of Science databases. Nine publications were included for the systematic review’s qualitative and quantitative analysis after the full-text screening, with five articles [9, 17–20] rejected based on eligibility criteria. Among the included papers, eight were cross-sectional studies, and one study was based on a retrospective quantitative methodology. Figure 1 shows a summarized flow chart detailing the article selection process.

### Quality of included studies

**Fig. 2:**
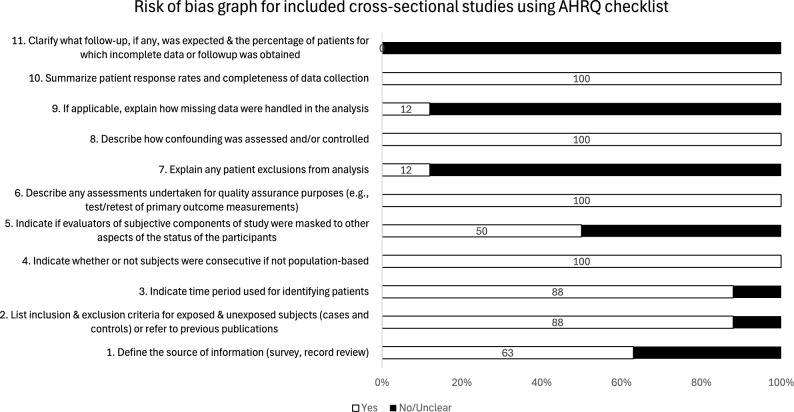
Risk of bias graph for included cross-sectional studies using the AHRQ checklist. The graph shows the risk of bias assessment for all included cross-sectional studies using the AHRQ tool. The y-axis represents the 11-checklist question, and x-axis represents the proportions of responses for each checklist question for all included cross-sectional studies. The white box depicts the percentage of studies that received a response “yes” for a checklist statement, and the black box shows the percentage of studies that received a “no” or “unclear” response for a checklist statement

Figure 2 presents the risk of bias assessment graph for all included cross-sectional studies in this systematic review. Of the 8 cross-sectional studies that were considered, 3 were of high quality, and 5 were of moderate quality. The quality assessment revealed that all the studies received a score of 1 for checklist statements numbers 6, 8, and 10. A score of 0 was given for statement number 11 because there was no follow-up evaluation following the baseline vision screening.

### Study characteristics

Nine papers were chosen for this systematic review after full-text screening, of which 8 prospective studies and 1 retrospective quantitative study were included. The overall sample size of the participants included in this systematic review ranged from 555 − 153,107. Four of the included studies reported that teachers received training in visual acuity testing as a one-day session [21–24]. Two other studies reported that teachers received training in a half-day session [25] and as a one-day workshop session [26]. In three studies [10, 11, 27], the number of training days or hours for teachers was not reported. Teachers in all the included studies received training from trained district education officers (DEOs) and eye care professionals. Teachers received training on measuring and documenting visual acuity, as well as information on the anatomy of the eye and common eye diseases during the training. Table 1 provides the study characteristics of all included papers.

**Table 1:** Included study characteristics

Study Author and Year Place	Age & Sample Size	Total teachers involved in screening	Tools used for vision screening	Comparison with the standard
Sharma A et al. 2008 [10] China	13–17 years *n* = 1893	32	Identical illuminated Snellen Tumbling E-vision chart at 6 m	Ophthalmologist/ Optometrist
Ostadimoghaddam H et al. 2012 [27] Iran	7–15 years *n* = 847	Not mentioned	Tumbling “E” LogMAR chart at 6 m	Optometrist
Teerawattananon K et al. 2014 [23] Thailand	4–12 years *n* = 5303	223	Lea symbol distance VA chart, “E” chart, Snellen chart at 6 m	Ophthalmologist
Saxena R et al. 2015 [11] India	6–15 years *n* = 9838	40	Modified ETDRS chart at 4 m	Primary eye care worker
Paudel P et al. 2016 [25] Vietnam	12–15 Years *n* = 555	14	LogMAR chart with tumbling-E optotypes at 6 m	Refractionists
Marmamula S et al. 2018 [21] India	4–15 years *n* = 3034	16	Test card with 6/12 tumbling E optotypes at 3 m	Vision technicians
Panda L et al. 2018 [22] India	5–15 years *n* = 153,107	216	Snellen E chart of 3 lines at 6 m	Optometrist
Tobi Pet al. 2022 [24] Liberia	5–18 years *n* = 823	8	Snellen chart at 3 m	Ophthalmic technicians
Sabherwal S et al. 2023 [26] India	6–17 years *n* = 3410	18	ETDRS type of LogMAR chart at 4 m	Optometrist

The table shows data extracted from nine included studies, which include author names, year of publication, age group, sample size, teachers involved, and tools used

The test reliability values from all included studies were extracted. Three investigations reported the TP, FN, FP, and TN. The RevMan calculator was used to estimate TN, FN, TP, and FP for the remaining 6 articles based on information about the total number of students examined and the proportion of people who had the target condition. The data on the sensitivity and specificity of vision screening conducted by teachers were extracted from all the included articles, and these values were reported for each specific cut-off criterion. Table 2 shows the test reliability values of vision screenings conducted by teachers compared with those of vision screenings conducted by eye care professionals.

**Table 2:** Sensitivity and specificity values from all included studies

Author	Year	Cut-off Criteria	TP (*n*)	FP (*n*)	FN (*n*)	TN (*n*)	*N*	Sensitivity	Specificity
Sharma et al.1	2008	UCVA ≤ 6/12	922	80	64	827	1893	0.93	0.91
Sharma et al.2	2008	PVA ≤ 6/12	534	192	93	1073	1892	0.85	0.85
Ostadi Moghaddam et al.	2012	< 6/9	21	63	35	728	847	0.37	0.92
Teerawattananon et al.1	2014	< 6/12 Pre-Primary school students	151	94	453	4604	5302	0.25	0.98
Teerawattananon et al.2	2014	< 6/12 Primary school students	357	94	248	4604	5303	0.59	0.98
Saxena et al.1	2015	< 6/9.5	1138	560	299	7841	9838	0.79	0.93
Saxena et al.2	2015	< 6/12	676	264	202	8696	9838	0.77	0.97
Saxena et al.3	2015	< 6/15	373	82	302	9081	9838	0.55	0.99
Paudel et al.1	2016	UCVA ≤ 6/12	117	18	18	402	555	0.86	0.95
Paudel et al.2	2016	PVA ≤ 6/12	73	32	24	426	555	0.75	0.93
Panda et al.	2018	< 6/9	66,564	32,897	16,114	37,532	153,107	0.80	0.53
Marmamula et al.	2018	< 6/12	44	3	17	2970	3034	0.72	0.99
Tobi et al.	2022	Could not see 3 out of 5 optotypes of 6/9 line	6	3	18	796	823	0.25	0.99
Sabherwal et al.1	2023	< 6/9.5 (ACTs)	91	123	162	3034	3410	0.36	0.96
Sabherwal et al.2	2023	< 6/9.5 (STs)	105	244	132	2518	2999	0.44	0.91

Table showing the cut-off criteria used in each study and the sensitivity and specificity values of each included study

*UCVA* Uncorrected Visual Acuity, *PVA* Presenting visual acuity, *TP* True positive, *TN* True negative, *FN* False negative, *FP* False positive, and *N* sample size, *ACTs* All class teachers, *STs* Selected school teachers

### Diagnostic accuracy meta-analysis

**Fig. 3:**
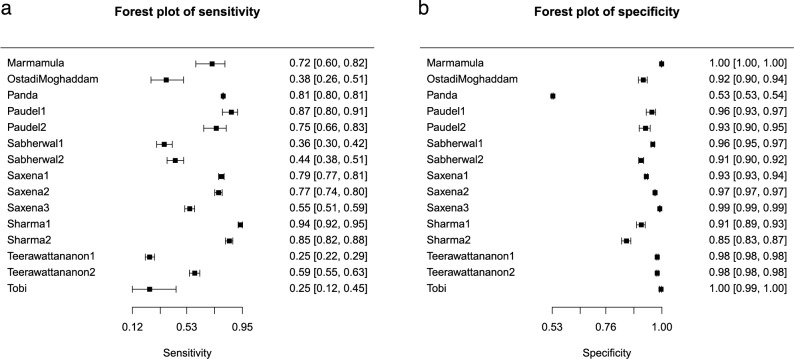
Forest plot of sensitivity and specificity for all included studies. **a** Shows a forest plot of pooled sensitivity values, and **b** shows pooled specificity values of vision screening performed by teachers from nine included studies. Each solid square corresponds to an individual study, with error bars indicating the 95% confidence intervals

To determine the pooled sensitivity and specificity, a forest plot analysis was performed using MetaDTA. In Fig. 3, the sensitivity and specificity values of vision screening conducted by school teachers are presented, encompassing the data from the nine included studies. From the forest plot (Fig. 3b), we can infer that the specificity of vision screening conducted by school teachers was ≥ 0.85 except for the study by Panda et al. [22]. The specificity values reported by Panda et al. were poor, as teachers felt unprepared to miss students with impaired vision, leading to unnecessary referrals of many children with normal vision, thereby negatively impacting the reliability of the screening results. From the forest plot (Fig. 3a), it is evident that the sensitivity was quite varied, ranging from 0.25 to 0.94, and 5 out of 9 studies had a sensitivity of less than 50%. The low sensitivity values in the Teerawattananon et al. study were linked to pre-primary school teachers, who found that vision screening for very young children was complex and time-consuming, requiring at least two individuals for vision screening due to the need for patience. Furthermore, Tobi et al. found that a lack of interest and motivation among teachers to conduct vision screening significantly affected the accuracy of the results.

**Fig. 4:**
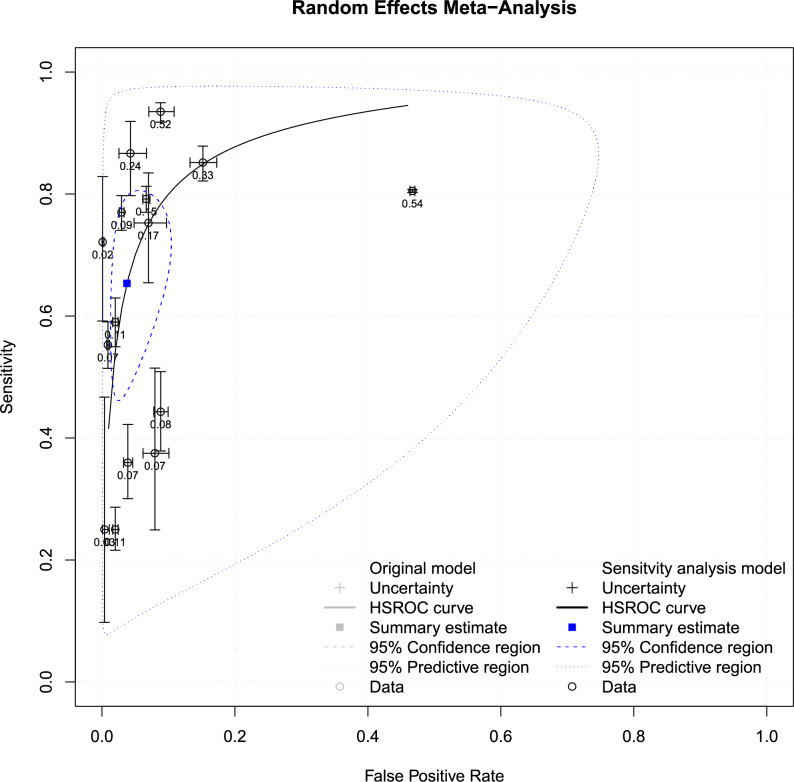
The SROC curve for the vision screening test performed by teachers among school- going children. The sensitivity levels are depicted on the y-axis, and the false positive rate values are represented on the x-axis in the graph. Each black unfilled circle illustrates the true positive rate (TPR) versus the false positive rate (FPR) for individual studies. The blue dotted circle delineates the predictive region encompassing all nine included studies, while the blue dotted line circle outlines the 95% confidence region for overall sensitivity and FPR. The comprehensive summary estimate for vision screening conducted by teachers is indicated by the square blue dot

To determine the diagnostic efficacy of the test, we used a summary receiver operating characteristic (SROC) curve approach. In this systematic review, SROC curve analysis was considered due to the availability of sensitivity and specificity data from multiple studies. The SROC was used to assess the overall diagnostic accuracy of the vision screening test by combining sensitivity and specificity values from multiple studies and achieving a consolidated summary estimate. The true positive rate (TPR) and false positive rate (FPR) of a vision screening test administered by teachers are plotted on the SROC curve. The total summary estimate of the diagnostic accuracy of vision screening by school teachers is depicted on the SROC curve in Fig. 4. The SROC curve analysis revealed overall sensitivity and specificity values of 0.65, 95% CI [0.51, 0.76] and 0.96, 95% CI [0.92, 0.98] respectively, for all nine included studies.

**Fig. 5:**
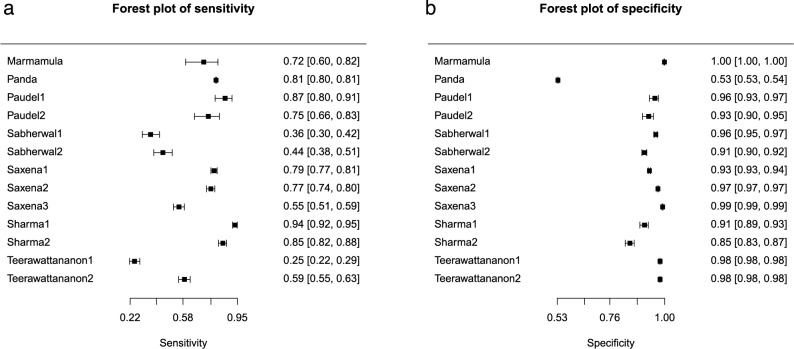
Forest plot of the sensitivity and specificity values excluding two studies. **a** shows a forest plot of pooled sensitivity values and **b** shows pooled specificity values of vision screening performed by teachers, excluding two studies from the analysis. Each solid square corresponds to an individual study, with error bars indicating the 95% confidence intervals

**Fig. 6:**
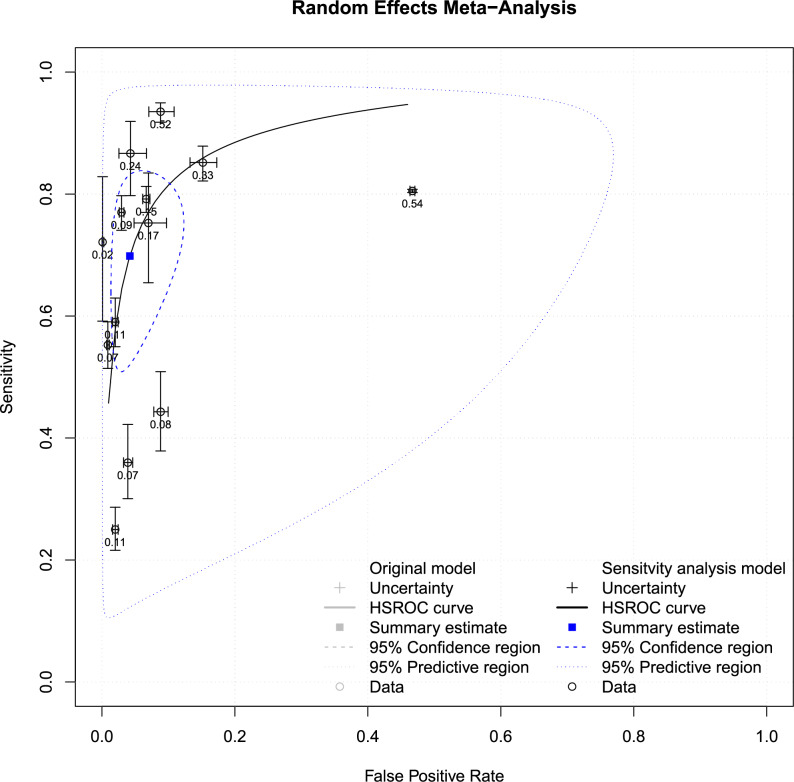
Reanalysis of the SROC curve for the vision screening test performed by teachers among school-going children. The SROC curve of vision screening tests performed by teachers, excluding two studies. Each black unfilled circle illustrates individual study estimates. The blue dotted circle delineates the predictive region by excluding two studies, while the blue dotted line circle outlines the 95% confidence region for overall sensitivity and FPR. The summary estimate for vision screening conducted by teachers is indicated by the square blue dot

The pooled analysis of all nine included studies revealed good specificity and moderate sensitivity. In the sensitivity plot, the study conducted by Ostadimoghaddam et al. [27] and Tobi et al. [24] reported poor sensitivity along with a wider CI range, signifying a larger variability in sensitivity estimation [28]. Therefore, a reanalysis was conducted by excluding the two studies [24, 27], and Fig. 6 shows the SROC curve plot. Figure 5 presents the forest plot depicting the sensitivity (5a) and specificity (5b) values of vision screening conducted by teachers, and the SROC curve plot is provided as Fig. 6, excluding those two studies. The reanalysis excluding two studies resulted in improved pooled sensitivity and specificity values of 0.70 (95% CI [0.57, 0.80]) and 0.96 (95% CI [0.91, 0.98]), respectively, for the vision screening conducted by teachers among school students.

## Discussion

This systematic review highlighted the effectiveness of vision screening performed by trained teachers in comparison with vision screening conducted by eye care professionals in the same setting. Nine publications were included as per the systematic review inclusion criteria, and after excluding two studies with greater variability in sensitivity, it resulted in the overall sensitivity of 0.70 (95% CI [0.57, 0.80]) and the specificity of 0.96 (95% CI [0.91, 0.98]).

In all the studies included in this systematic review, eye care professionals had provided training to teachers on how to measure visual acuity (VA), record VA, use a VA chart, and record data on a data form [25, 26]. The training session conducted for teachers also provided information on prevalent eye conditions affecting school-age children [26]. Also, teachers were assigned a specific visual acuity cut-off point to refer the students for a higher assessment.

In this systematic review, the quantitative analysis revealed that vision screening conducted by teachers had good specificity for vision screening, on par with that of eye care experts. This indicates that school teachers more accurately identified children with normal eyesight. In this systematic review, one [22] of the included studies reported poor specificity for teacher-conducted vision screening among school children. This could be because of the stricter criteria (< 6/9) using a 3-line Snellen chart used in this study, compared to the majority of the studies where the screening cut-off was 6/12. The poor specificity of vision screening by teachers was reported to be associated with inadequate teacher supervision, training, and lower levels of motivation due to workload, as well as poor screening methods [29]. Panda et al. noted that the one-day training provided to teachers and teachers unfamiliarity with the screening program, may have contributed to the observed poor specificity [22]. Furthermore, failing to test the presenting visual acuity with glasses among children who wear them may have also contributed to the loss of specificity in vision screenings conducted by school teachers [10]. Additionally, challenges in comprehension among younger children compared to older children have been identified as a contributing factor to lower specificity [20, 24]. The low specificity implies that the teachers were unwilling to take any risks in potentially missing cases of impaired vision [22]. A program for eye screening with low specificity would be ineffective and would not benefit the population being examined [10]. The pooled estimation of specificity in this investigation suggests that teachers were extremely precise in the detection of children with normal vision.

The pooled sensitivity of 7 included studies was estimated to be 0.70 (95% CI [0.57, 0.80]) for vision screening conducted by school teachers, based on comparisons with a reference standard screening conducted by eye care professionals in each included study. The factors that can affect sensitivity could be the screening cut-off criteria and the prevalence of the disease condition. A lenient cut-off might result in high false negatives, and a low prevalence might result in low true positives, which can affect sensitivity. For the studies included in this systematic review, the probable reason could be the variation in the prevalence of visual impairment, which ranged from 2% to 54%. The studies with poor sensitivity were noted to have a lower prevalence of visual impairment.

In this systematic review, there were studies that reported lower sensitivity, and one study reported poor specificity for vision screening performed by teachers in comparison with vision screening conducted by eye care professionals. The differences in terms of school demographics, screening procedures, the use of different charts, disease prevalence, and cut-off criteria could have attributed to these differences. Several factors, such as motivation, spectacle wear, employment in public or private schools, and years of teaching experience, can influence a teacher’s ability to accurately identify a problem [23]. In this review, we did not assess publication bias due to an insufficient number of studies. Despite this, teachers are adequately trained to conduct basic vision screenings in school-based programs, given their extensive knowledge and experience in working with children [10, 22]. The teachers accessibility, familiarity with students, and respected status in the community enable them to effectively engage families, encourage participation in vision screenings and follow-ups, and promote consistent use of spectacles [11, 22]. The literature suggests that the cut-off criteria for teacher-conducted vision screening should be distinct from those used by health professionals to enhance the effectiveness of the diagnosis [23]. To enhance the effectiveness of teacher-conducted vision screenings, there is a need for structured school vision screening protocols with periodic evaluation.

## Conclusion

The pooled sensitivity and specificity from 7 studies for teachers conducted vision screening were 0.70 (95% CI [0.57, 0.80]) and 0.96 (95% CI [0.91, 0.98]), respectively. These findings suggest that teachers can be a potential workforce to support eye care professionals in the preliminary vision screening. Also, factors such as the type of training provided to school teachers, the age of the screening population, the cut-off criteria used, and the prevalence of the ocular condition can significantly influence the effectiveness of teacher-led vision screening programs. Additionally, consistent adherence to protocols and meticulous data management are crucial for ensuring the accuracy of the screening results.

## Supplementary Information

Supplementary Material 1

## Data Availability

This article contains all the data that were analyzed during the study or extracted from published literature. Further enquiries can be directed to the corresponding author.

## Abbreviations

VI: Visual Impairment
SES: School Eye Screening
VA: Visual Acuity
UVA: Uncorrected Visual Acuity
BCVA: Best Corrected Visual Acuity
PVA: Presenting Visual Acuity
PRISMA: Preferred Reporting Items for Systematic Reviews and Meta-analyses
AHRQ: Agency for Healthcare Research and Quality
TP: True Positive
TN: True Negative
FP: False Positive
FN: False Negative
SROC: Summary Receiver Operating Characteristics
