# Supplementary material for: Effectiveness of school vision screening by teachers – a systematic review and meta-analysis
Source: BMC Public Health. 2026 Apr 20;26:1794. doi: 10.1186/s12889-026-27333-0 (PMC13235086; doi:10.1186/s12889-026-27333-0)
Supplement: Supplementary file 1 — Supplementary Material 1 [file 12889_2026_27333_MOESM1_ESM.docx]

**Supplementary material 1:** Search strategy and the number of articles retrieved

| Search string - Pubmed | Retrieved articles |
| --- | --- |
| ((((((vision screening) AND (school children)) OR (school-going children)) AND (trained teacher)) OR (teacher)) AND (eye care professional)) OR (optometrist) | 2852 |
| ((((((((vision screening[MeSH Terms]) OR (vision screenings[MeSH Terms])) OR (eye screening[MeSH Terms])) AND (school children[MeSH Terms])) AND (school childrens[MeSH Terms])) OR (school going children[MeSH Terms])) AND (school teacher[MeSH Terms])) OR (school teachers[MeSH Terms])) OR (trained teacher[MeSH Terms]) | 2569 |
| ((((((((school teacher[Title/Abstract]) OR (teacher[Title/Abstract])) OR (trained teacher[Title/Abstract])) AND (eye care specialist[Title/Abstract])) OR (eye care professional[Title/Abstract])) OR (optometrist[Title/Abstract])) OR (refractionist[Title/Abstract])) AND (vision screening[Title/Abstract])) OR (eye screening[Title/Abstract]) | 600 |
| ((((((((((school teacher[Title/Abstract]) OR (teacher[Title/Abstract])) OR (trained teacher[Title/Abstract])) AND (eye care specialist[Title/Abstract])) OR (eye care professional[Title/Abstract])) OR (optometrist[Title/Abstract])) OR (refractionist[Title/Abstract])) AND (vision screening[Title/Abstract])) OR (eye screening[Title/Abstract])) AND (sensitivity[Title/Abstract])) AND (specificity[Title/Abstract]) | 75 |
| ((((sensitivity) AND (specificity)) AND (vision screening)) AND (school children)) AND (school teacher) | 21 |

| Search string - Web of Science | Retrieved articles |
| --- | --- |
| vision screening | 925 |
| ((ALL=(vision screening)) AND ALL=(school)) AND ALL=(teachers) | 125 |
| (((TI=(school children)) AND TI=(eye screening)) OR TI=(vision screening)) OR TI=(teacher vision screening) | 932 |
| ((ALL=(school children)) AND ALL=(vision screening)) AND ALL=(school teachers) | 93 |
| ((((((AB=(vision screening)) OR AB=(eye screening)) OR AB=(visual screening)) AND AB=(school children)) OR AB=(school going children)) AND AB=(sensitivity)) AND AB=(specificity) | 85 |
| ((ALL=(sensitivity )) AND ALL=(vision screening)) AND ALL=(school children) | 176 |
| ((ALL=(specificity)) AND ALL=(vision screening)) AND ALL=(school children) | 147 |

| Search string - Scopus | Retrieved articles |
| --- | --- |
| vision AND screening AND school AND children AND teacher | 2451 |
| vision AND screening OR eye AND screening AND children OR school AND children OR school AND going AND children AND teacher | 484 |
| school AND children OR school AND going AND children AND vision AND screening OR eye AND screening AND trained AND teacher OR teachers AND optometris | 9 |
| eye AND care AND professional OR optometrist AND eye AND screening AND school AND children OR children AND trained AND teacher | 128 |
| trained AND teacher OR teacher OR school AND health AND care AND worker AND eye AND care AND professional OR optometrist OR eye AND care AND specialists AND eye AND screening AND school AND children OR children | 58 |
